# Experimental evolution reveals genomic signatures of variety-specific selection of *Cercospora beticola* in Germany

**DOI:** 10.1038/s41598-026-52994-7

**Published:** 2026-05-21

**Authors:** Yixuan Yang, Nathan A. Wyatt, Ana L. Martinez, Melvin D. Bolton, Britt-Louise Lennefors, Harald Keunecke, Heinrich Reineke, Maria Köhler, Mark Varrelmann, Sebastian Liebe

**Affiliations:** 1 Institute of Sugar Beet Research, Göttingen, Germany; 2 Sugarbeet Research Unit, USDA-ARS, Fargo, ND USA; 3 Department of Plant Pathology, North Dakota State University, Fargo, ND USA; 4 DLF Beet Seed AB, Landskrona, Sweden; 5 KWS SAAT SE & Co. KGaA, Einbeck, Germany; 6 SESVanderHave Deutschland GmbH, Würzburg, Germany; 7 Strube D&S GmbH, Söllingen, Germany

**Keywords:** Genetics, Microbiology, Plant sciences

## Abstract

Cercospora leaf spot, caused by *Cercospora beticola*, is the most destructive foliar disease of sugar beet. Management has relied heavily on fungicides, but widespread fungicide resistance emphasized the need for resistant varieties. To assess how varietal resistance shapes *C. beticola* populations, we conducted a 3-year field experiment at four locations in Germany using sugar beet varieties ranging from susceptible to highly resistant. Infected leaves were collected separately per variety and location to serve as inoculum for the following year, maintaining variety- and location-specific pathogen populations under controlled selective conditions. A total of 900 isolates were subjected to whole-genome sequencing and population genetic analyses. Isolates collected in 2022 showed extensive admixture among locations. Equal mating-type ratios were observed in some populations but skewed in others, suggesting that sexual reproduction may occur locally. Location-specific re-inoculation preserved population continuity but did not produce detectable local adaptation. By contrast, the population was affected to some extent by variety-specific re-inoculation. Populations from one highly resistant variety formed two divergent lineages with reduced diversity and altered allele frequency spectra, consistent with host-driven selection. Genome scans identified 26 candidate genes, including two putative effectors, associated with adaptation to the highly resistant variety. These findings demonstrate rapid *C. beticola* adaptation to resistant varieties, highlighting the role of host-driven selection in resistance management.

**Supplementary Information:**

The online version contains supplementary material available at 10.1038/s41598-026-52994-7.

## Introduction

Fungal pathogens cause diseases that challenge plant, animal, and human health^1^. Fungi are known for their rapid adaptation in various environments facilitated by high genomic plasticity and the ability to leverage mixed sexual and asexual reproduction modes^2–5^. Under global climate change, the emergence of new plant diseases caused by fungal pathogens threatens the modern agricultural system^6–9^. An agroecosystem is described as a genetically uniform environment where the pathogens are highly dependent on the host. Under this environment, selection pressure is highly specific compared to the natural ecosystem, hence, rapid adaptation of pathogens can be expected^10,11^. Various strategies are employed to ensure a stable crop yield, including agronomic practices and the application of fungicides. The most sustainable approach is the implementation of resistant varieties. However, the introduction of a resistant variety offers a stressful environment for fungal populations that often leads to a high pressure of directed selections^11^. Evolved adaptation allows fungi to overcome resistance, especially for those who do not have a strict asexual reproductive mode^3,4,10^. Hence, understanding the adaptation and evolutionary events within a fungal population is crucial for developing more effective management strategies.

Population genetics is a powerful approach for studying the transmission, reproduction, and evolutionary processes of filamentous fungi^12–14^. By unveiling population structures of plant pathogenic fungi, we can develop a greater understanding of the genetic variation and spatiotemporal dynamics of evolution. For example, introducing a new resistant varieties can generate positive selection that alters the evolutionary trajectory of pathogen populations. This leaves signatures of selection across pathogens’ genomes that can be detected using advanced population genetic tools^15,16^. One commonly used measure is Tajima’s D^17^, in which mean pairwise differences and the number of segregating sites are compared to test a null hypothesis of mutation-drift equilibrium and constant population size^14,18^. A negative Tajima’s D, for example, reflected by elevated segregating sites, is interpreted as a signature of populations under selection. Furthermore, advances have been achieved in detecting signatures of selection genome-wide, termed selective sweeps, which exhibit reduced variation and inflated linkage disequilibrium (LD)^14,18^.

Cercospora leaf spot (CLS) is considered the most destructive foliar disease in sugar beet worldwide, which can cause dramatic reductions in sugar yield^19–21^. The haploid fungal pathogen *Cercospora beticola* is the causal agent of CLS^22^. Symptoms of CLS appear as dark-centred lesions with dark-reddish rings on leaves and petioles that are the typical morphological factor to distinguish between CLS and other foliar diseases^22–24^. The polycyclic lifestyle of *C. beticola* enables multiple infection cycles in one season, which reinforces the severity of CLS on sugar beet^21,25^. The primary infectious material is thought to be conidia derived from overwintered structures that survive in plant debris^26^. However, this view was challenged by population genetic studies showing high admixture and weak geographic differentiation among *C. beticola* populations, suggesting that epidemics could also be contributed by regionally mixed inoculum rather than strictly local sources^27,28^. After the success of the initial infection cycle, sporulation may occur again from pseudostromata that formed in lesions. Spores are dispersed by wind, rain splash, and human activity to neighbouring hosts and fields^22,25^.

Sugar beet (*Beta vulgaris*. ssp. *vulgaris*) has a relatively short domestication history but is responsible for major sugar production in the northern hemisphere, as well as serving in biogas and animal feed production^22,29^. Conventional breeding efforts have been undertaken in sugar beet to incorporate various forms of resistance to CLS^29^. Monogenic resistance in sugar beet against CLS was reported in the late twentieth century but soon abolished due to its instability^29,30^. Wild relatives of sugar beet are used as genetic resources to explore novel resistance against *C. beticola*. For instance, sea beet (*B. vulgaris* subsp. *maritima*) has been sourced for disease resistant traits for sugar beet breeding^31–33^. Presently, resistant varieties are available, which cannot completely prevent infection but dramatically reduce disease development^34,35^.

Genetic studies have revealed that *C. beticola* populations possess high genotypic diversity globally, with temporal shifts in selected populations and distinct genetic differentiation between certain regions. For example, New Zealand populations are significantly divergent from European populations, and New York isolates show divergence from other regions^3,4,27,28,36,37^. This diversity is considered to be indirect evidence of regular sexual recombination, although no discernible teleomorph of *C. beticola* has been identified up-to-date^3,4,27^. The high genotypic diversity may also grant *C. beticola* the capability to adapt to new environments and overcome host resistance relatively quickly^11,38,39^. The increased frequency of fungicide resistance in *C. beticola*^3,4,40–42^ is an example of *C. beticola*’s high potential for adaption. With the concern of increasing fungicide resistance of *C. beticola*, more effort has been drawn to implement resistance in sugar beet cultivation. Although the population structure of *C. beticola* from various host species has been analyzed previously^36,37^, no examination of how *C. beticola* population structure can be shaped by different host varieties of the same species has been performed.

This study aimed to investigate the genetic diversity and structure of *C. beticola* populations from selected geographical locations in Germany derived from sugar beet varieties with varying levels of CLS resistance. For this purpose, we analyzed a total of 900 *C. beticola* isolates derived from four sugar beet varieties with different resistance properties. The collection of samples was performed over three consecutive years in four geographic locations in Germany. To accelerate the adaptation process, fields were re-inoculated each season using inoculum derived from the previous year’s infections, collected separately from each location and variety, thereby generating strong selective pressure. With this approach, we were able to observe a rapid change in the genetic diversity and structure of *C*. *beticola* populations in a host-driven selection process.

## Results

### Genomic data

After processing the raw sequencing data, we retained a total of 900 sequences from isolates from four host varieties across four locations in Germany over three sampling years (see Supplementary Table S1 online). Among these isolates, the average genome coverage was 24.5× (see Supplementary Table S2 online). Variant calling identified 1,431,483 SNPs that were further filtered to a final set of 733,351 high confidence SNPs.

### Baseline structure of *C*. *beticola* populations in Germany before re-inoculation

**Fig. 1:**
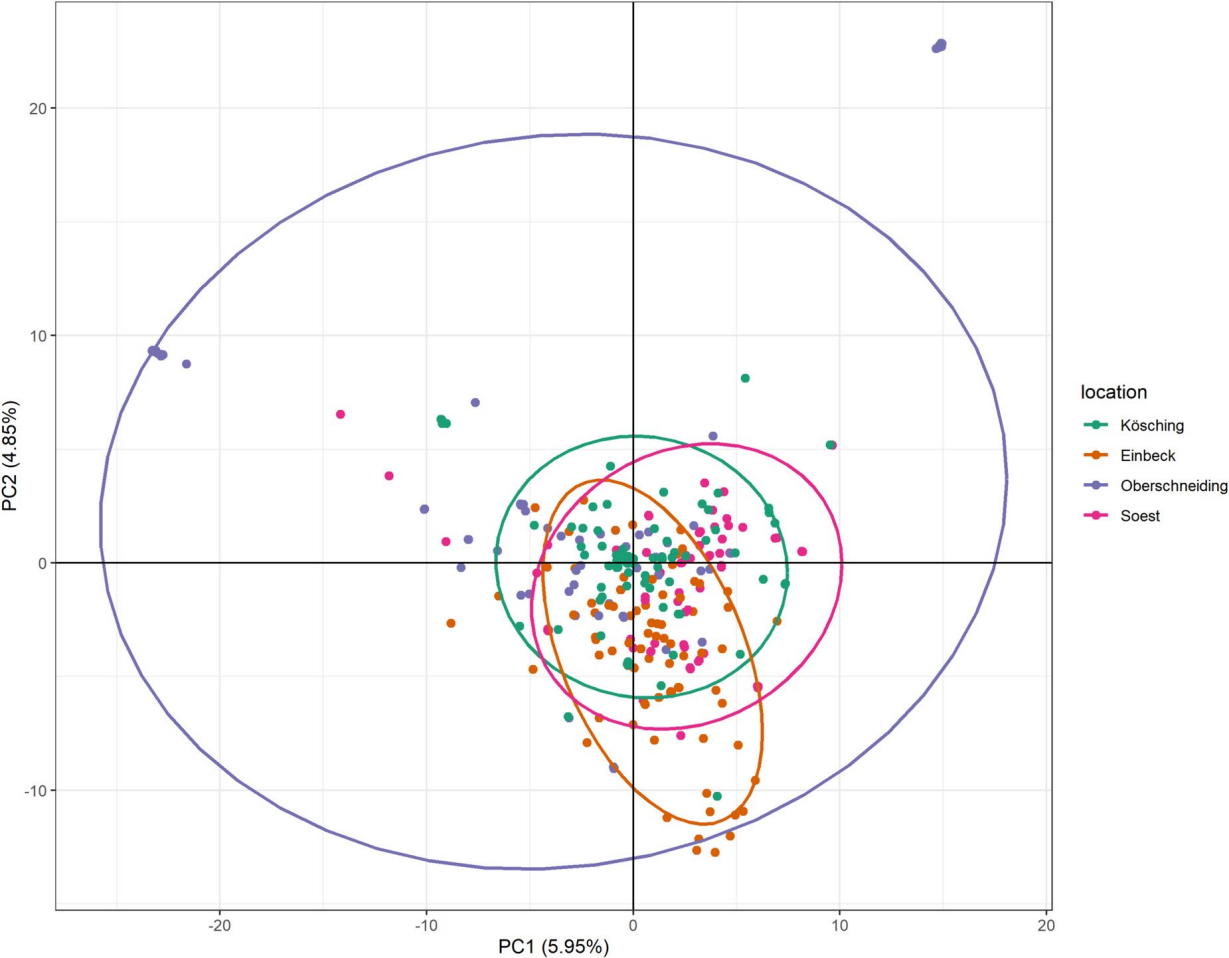
PCA of *C. beticola* isolates collected in 2022 from four trial locations in Germany. Different colors represent the sampling locations, and ellipses indicate the 95% confidence interval of each population.

We examined the population structure from isolates collected in 2022 using an independent SNP dataset to establish a population genetic baseline prior to re-inoculation. Our PCA result (Fig. 1) suggests that *C. beticola* populations in Germany have a minor geographical structure with major admixture of populations from all four locations. Several location-specific lineages could be identified from Oberschneiding. Isolates from Oberschneiding had the highest within-location divergence, while isolates from other locations were more homogeneous.

We further performed an AMOVA on the same dataset to better understand the source of variation in the population. The results (Table 1) showed that most variation was identified between individuals, accounting for 92.7% of genetic variation. In addition, a fraction of 4.8% of the variation is attributed to variation between locations and less than 3% of the variance could be explained among samples from different host varieties within locations. All components of variance were statistically significant (*p* < 0.001). Overall, the AMOVA results suggest that population variation is primarily contributed by differences between individuals with a minor but significant geographic structure of the *C. beticola* population in Germany.

**Table 1:** AMOVA results of populations from four locations in Germany in 2022.

Variation	Df	Sum sq	%	*p*
Between locations	3	15107.35	4.8	0.001
Between samples within locations	12	15661.85	2.5	0.001
Between samples	329	272649.28	92.7	0.001
Total	344	303418.45	100	

Furthermore, we investigated the population-wise nucleotide diversity (π) to compare genetic diversity, which can help to infer the recent demographic history of the populations. Across all populations, π values ranged from 0.00370 to 0.00394 (Table 2). Although the values were similar, a significant difference in nucleotide diversity was detected among populations from different locations (Kruskal–Wallis, *p* = 0.03029). We further examined the differences in each pair of populations (Wilcoxon rank sum test). Of all the populations, the one from Oberschneiding showed significantly lower genetic diversity than the ones from Kösching and Soest. However, no significant difference was observed compared to the population from Einbeck. Meanwhile, the populations from Kösching, Einbeck, and Soest exhibited no difference in π.

**Table 2:** Nucleotide diversity and Tajima’s D of populations from four locations in 2022. Significant differences are indicated by small letters based on Wilcoxon rank sum test.

Location	π (*p* = 0.03029)		Tajima’s D (*p* = 1.532e^-6^)	
	Value	Standard deviation	Value	Standard deviation
Kösching	0.003943569^a^	0.005212197	0.3808742^ab^	1.490474
Einbeck	0.003791756^ab^	0.004896440	0.3905639^b^	1.589617
Oberschneiding	0.003704874^b^	0.004994746	0.2668955^c^	1.666403
Soest	0.003888963^a^	0.005138275	0.4481962^a^	1.517194

We further compared the values of Tajima’s D to gain better insight into the recent demographic history of each population (Table 2). Overall, all populations showed positive Tajima’s D values that deviate little from zero, suggesting balancing selection of the German *C. beticola* populations. Significant differences in the population-wise Tajima’s D were observed between the four population locations (Kruskal–Wallis *p* value < 0.05). The additional pairwise Wilcoxon rank sum test revealed that the population in Oberschneiding showed a significantly lower Tajima’s D than other populations, aligning with the lowest level of nucleotide diversity.

*C. beticola* is a heterothallic fungal pathogen; therefore, the distribution of the two mating type genes (*MAT1-1-1* and *MAT1-2-1*) can be used to infer the reproduction mode of populations. Hence, we calculated the relative frequency of each mating type gene in the populations (see Supplementary Fig. S1 online). Our results showed a significant divergent from a 1:1 distribution of two mating types in populations from Kösching and Soest and non-significant results in populations from Einbeck and Oberschneiding.

### Temporal dynamics of *C. beticola* population structure

**Fig. 2:**
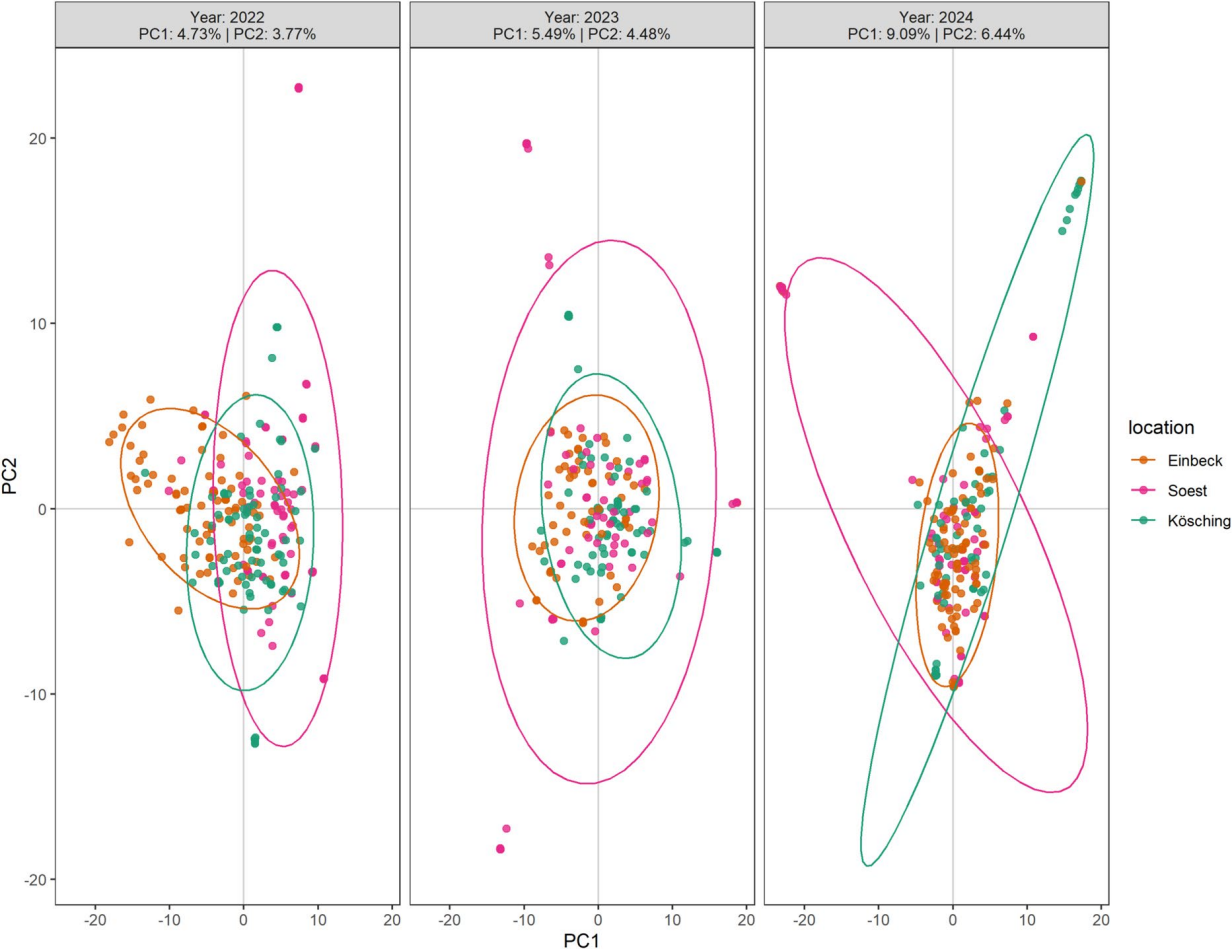
PCA of *C. beticola* isolates collected from three re-inoculated locations across 3 years. Different colors represent the sampling locations, and ellipses indicate the 95% confidence interval of each population.

To gain insight into the temporal dynamics of population structure at each location, we analyzed populations collected over 3 years. Due to the low infestation pressure in Oberschneiding in 2022, no inoculum could be collected at this location to continue further re-inoculation trials in the next 2 years. Therefore, we had to exclude this location from the study in 2023 and 2024. To initially assess whether location-specific re-inoculation maintained populations across 3 years, we constructed distance trees based on pairwise bitwise genetic distance (see Supplementary Figs. S2–S4). Isolates from different years were interspersed within each location rather than forming year-specific clusters. This suggests that populations from each location originated from the same genetic pool. To address the effect of location-specific re-inoculation on the population structure, we performed a PCA for each location over 3 years (Fig. 2). Both PC1 and PC2 increased over 3 years, indicating increased dispersion of individuals within each location. In 2022, we do not see apparent divergence among locations. In 2023, the population from Soest exhibited increased dispersion compared to the other two populations, while the other two populations appeared to have less separation. After another year of re-inoculation, we observed more distinct location-associated clusters from Soest and Kösching in 2024, while the population from Einbeck remained relatively central and compact. These patterns coincide with AMOVA results that showed continuously increased variation among isolates within locations (see Supplementary Table S3 online). This result also hinted at an early increased divergence of isolates within the same location, which aligned with the PCA.

To better understand the features of each population from different locations, we then calculated the π and Tajima’s D (Table 3) of each population. In 2022, no significant differences were revealed across populations in both π and Tajima’s D (Kruskal–Wallis test; *p*_π_ = 0.3097, *p*_D_ = 0.985). This result aligned with our observation in the PCA, where the clusters largely overlapped. After 1 year of location-specific re-inoculation, no significant difference was observed in π (*p*_π_ = 0.7434), while a significant difference was observed in Tajima’s D among populations (*p*_D_ < 0.001). A significantly lower Tajima’s D in the population from Kösching was observed compared to the other populations (Wilcoxon rank sum test, *p* < 0.001). Moreover, compared to 2022, only this location showed a reduced Tajima’s D value, while the other two populations had an increase.

**Table 3:** Nucleotide diversity and Tajima’s D of *C*. *beticola* populations collected from three re-inoculated locations across 3 years. Significant differences were determined by post-hoc pairwise Wilcoxon tests (*p* < 0.05). SD indicates standard deviation.

Year	Location	Mean	Median	SD	n	Letter
Nucleotide diversity						
2022	Einbeck	0.00379	0.00170	0.00490	3523	a
2022	Kösching	0.00394	0.00176	0.00521	3523	a
2022	Soest	0.00389	0.00172	0.00514	3523	a
2023	Einbeck	0.00399	0.00174	0.00524	3523	a
2023	Kösching	0.00396	0.00174	0.00528	3523	a
2023	Soest	0.00395	0.00170	0.00530	3523	a
2024	Einbeck	0.00393	0.00178	0.00519	3523	b
2024	Kösching	0.00369	0.00154	0.00506	3523	a
2024	Soest	0.00388	0.00168	0.00517	3523	b
Tajima’s D						
2022	Einbeck	0.39056	0.25301	1.58962	3521	a
2022	Kösching	0.38087	0.27908	1.49047	3520	a
2022	Soest	0.44820	0.34908	1.51719	3520	a
2023	Einbeck	0.46124	0.37186	1.46548	3519	b
2023	Kösching	0.26526	0.13409	1.51873	3519	a
2023	Soest	0.49413	0.40511	1.49300	3521	b
2024	Einbeck	0.40799	0.26984	1.53010	3522	b
2024	Kösching	0.24526	0.10516	1.53924	3520	a
2024	Soest	0.53758	0.43011	1.52115	3520	c

After the last re-inoculation in 2024, significant differences among populations were observed in both π and Tajima’s D (Kruskal–Wallis test; *p*_π_ = 0.0013, *p*_D_ = 6.322e^−16^). A pairwise Wilcoxon test showed a significantly lower diversity of the population from Kösching. In addition, we observed a continuous decrease in Tajima’s D compared to the past 2 years in the same population, pinpointing the selection that drove the within-population divergence. Conversely, Tajima’s D in population Soest continuously increased throughout 3 years, while the diversity remained stable, suggesting an excess of intermediate-frequency alleles. This result is supportive to our PCA observation where a location-associated lineage was formed in 2024. Meanwhile, the Einbeck population showed genetic stability and consistent allele frequency distribution (Table 3), which aligned with the centrally clustered population in the PCA throughout 3 years.

### Effect of variety-specific re-inoculation on host-driven population structure

A key aspect of our study was to address the effect of variety resistance on the *C. beticola* population. For this purpose, we performed variety-specific re-inoculation over 3 years at each location, aiming to assess whether strong selective pressure occurs on each variety-specific population (see Supplementary Fig. S5 online). Among all locations where the re-inoculation was successful, we grouped the isolates based on their host variety to conduct analysis. Disease severity assessments across 3 years consistently differentiated the four sugar beet varieties (see Supplementary Fig. S6 online). Variety A displayed the highest disease severity in all environments, confirming its susceptibility, whereas variety D consistently showed the lowest severity, reflecting its high resistance. Varieties B and C exhibited intermediate levels of disease severity, with B generally higher than C. The pattern was generally stable across locations and over the years. Differentiation between varieties was lowest in 2024, after the second reinoculation took place.

**Fig. 3:**
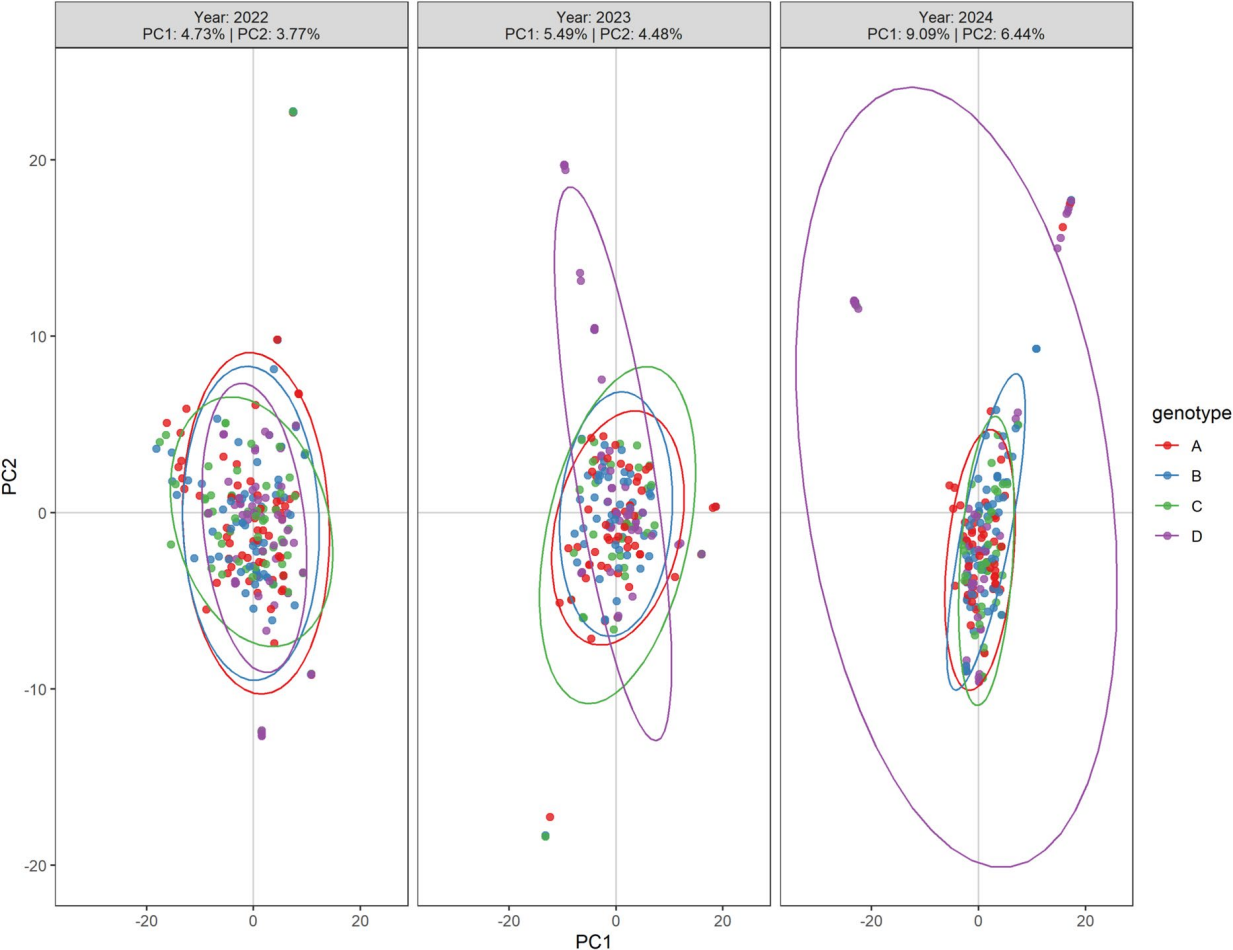
PCA of *C. beticola* isolates collected from four sugar beet varieties across 3 years. Different colors represent the host variety, and ellipses indicate the 95% confidence interval of each population.

A PCA was first performed to describe the population structure over 3 years of variety-specific re-inoculation (Fig. 3). In 2022, isolates collected from all four varieties did not show separation of clustering. Instead, we observed a substantial overlap across isolates collected from different varieties, suggesting minimal population differentiation. This result was reinforced by AMOVA (see Supplementary Table S4 online), where no significant variation was explained by the between-variety factor in 2022 (*p* value: 0.982). The minor but significant fraction of the variation that is explained by the factor between individuals within the population (5.53%, *p* value: 0.001) further supported our observation from the PCA.

After the first variety-specific re-inoculation in 2023, we observed higher values in both PC1 and PC2, suggesting an overall increase in genetic dispersion (Fig. 3). However, although an increased percentage of variation between host varieties was observed in AMOVA (see Supplementary Table S4 online), the large *p* value suggested no significant effect of this factor. While populations from varieties A, B, and C remained near the PCA center with similar or slightly increased within-population dispersion, the population from variety D exhibited substantial dispersion, spreading extensively along PC2. Moreover, this observation also aligned with an increased within-population variation (6.04%, *p* = 0.001) in AMOVA. These results suggested a pronounced genetic response of these isolates. In 2024, the population from variety D formed new variety-associated clusters distant from the core cluster. AMOVA showed a further increase in within-population variation in 2024 (8.86%, *p* = 0.001). These results suggested an increased divergence within the population collected from variety D, indicating an effect of variety-specific re-inoculation specifically on the corresponding population.

Moreover, to further investigate genetic variation and potential signatures of selection in each *C. beticola* population from different varieties, we calculated π and Tajima’s D for each population each year (Table 4). In 2022, there were no significant differences in nucleotide diversity among the populations from different sugar beet varieties, which aligns with our PCA results where clusters largely converged. We identified significant differences among populations in both 2023 and 2024 (*p* value 0.017 and 0.002, respectively). In both 2023 and 2024, the population from variety D showed significantly lower π than other populations, suggesting a decreased within-population diversity, potentially indicating direct selection in this population. This was also supported by our Tajima’s D results, in which the population from variety D showed an increase over the 3 years compared to the populations sampled from the varieties. In both years after the variety-specific re-inoculation, the population from variety D exhibited a significantly higher Tajima’s D value compared to other populations. Taken together, these patterns indicate that the recurrent re-inoculation on the resistant variety D imposed selective pressure, leading to reduced genetic diversity and a shift in allele frequency within its *C. beticola* population. Pairwise Fst (fixation index) comparisons (Table 5) revealed almost no differentiation among populations in 2022, consistent with the PCA and AMOVA results showing high overlap among populations. In 2023 and 2024, however, differentiation between the population collected from variety D and other populations increased, whereas populations collected from other varieties remained undifferentiated. The robustness of the mean pairwise Fst estimates is further supported by confidence intervals calculated by bootstrap resampling.

**Table 4:** Nucleotide diversity and Tajima’s D of *C*. *beticola* populations collected from four re-inoculated varieties across 3 years. Significant differences were determined by post-hoc pairwise Wilcoxon tests (*p* < 0.05). SD indicates standard deviation.

Year	Location	Mean	Median	SD	n	Letter
Nucleotide diversity						
2022	A	0.00395	0.00179	0.00513	3523	a
2022	B	0.00399	0.00183	0.00513	3523	a
2022	C	0.00398	0.00179	0.00513	3523	a
2022	D	0.00391	0.00172	0.00513	3523	a
2023	A	0.00403	0.00182	0.00526	3523	a
2023	B	0.00409	0.00180	0.00541	3523	a
2023	C	0.00403	0.00173	0.00535	3523	ab
2023	D	0.00391	0.00159	0.00529	3523	b
2024	A	0.00401	0.00180	0.00530	3523	a
2024	B	0.00392	0.00168	0.00530	3523	ab
2024	C	0.00394	0.00177	0.00518	3523	a
2024	D	0.00384	0.00157	0.00530	3523	b
Tajima’s D						
2022	A	0.47328	0.37956	1.48383	3523	a
2022	B	0.27686	0.17719	1.44785	3523	b
2022	C	0.37854	0.26142	1.46224	3523	ac
2022	D	0.31399	0.19418	1.49709	3523	bc
2023	A	0.21903	0.09317	1.44956	3523	a
2023	B	0.30487	0.15451	1.43761	3523	ab
2023	C	0.31298	0.24079	1.37642	3523	b
2023	D	0.57819	0.53154	1.44932	3523	c
2024	A	0.37410	0.24691	1.45136	3523	ab
2024	B	0.29683	0.14418	1.49609	3523	a
2024	C	0.42283	0.33913	1.39608	3523	b
2024	D	0.59502	0.59382	1.61248	3523	c

**Table 5:** Pairwise Fst of populations from different variety across 3 years including standard deviation (SD), lower (LCL) and upper (UCL) confidence limits.

Year	Population 1	Population 2	Fst	SD	LCL	UCL
2022	A	B	0.00074	0.00405	0.00061	0.00089
2022	A	C	0.00111	0.00556	0.00094	0.00130
2022	A	D	0.00765	0.01729	0.00703	0.00825
2022	B	C	0.00127	0.00591	0.00107	0.00146
2022	B	D	0.00780	0.01944	0.00719	0.00845
2022	C	D	0.00565	0.01447	0.00520	0.00613
2023	A	B	0.00178	0.00843	0.00151	0.00208
2023	A	D	0.01648	0.03151	0.01541	0.01757
2023	B	D	0.01263	0.02680	0.01178	0.01349
2023	C	A	0.00278	0.01124	0.00242	0.00318
2023	C	B	0.00153	0.00808	0.00128	0.00180
2023	C	D	0.01693	0.03514	0.01577	0.01813
2024	A	B	0.00845	0.02484	0.00765	0.00926
2024	A	D	0.01782	0.03799	0.01653	0.01898
2024	B	D	0.02109	0.04320	0.01968	0.02254
2024	C	A	0.00242	0.01068	0.00206	0.00275
2024	C	B	0.00530	0.01859	0.00471	0.00588
2024	C	D	0.01740	0.03689	0.01621	0.01861

### Identification of key regions for divergence and selective sweeps

In our 3-year study, sugar beet variety A consistently showed the highest disease severity, whereas variety D exhibited mostly the lowest severity values across all four trial locations. Therefore, we assume the population from variety A had undergone the least selective pressure through the variety-specific re-inoculation practices. In contrast, the population collected from variety D seemed to react to the selective pressure imposed by this variety. Hence, we chose the populations from these two varieties to identify key regions showing the most divergence.

For this purpose, we calculated genome-wide Weir and Cockerham Fst and Dxy using a fixed window size of 10 Kb. To identify the most divergent regions, we selected regions in the top 5% value in both statistics and overlapped them. In 2024, we identified nine highly divergent regions (see Supplementary Table S5 online). Furthermore, we deployed RAiSD to detect regions showing signatures of selective sweeps in the population collected from the resistant variety D in 2024, which identified 224 regions. By overlapping the most divergent regions with regions that exhibited signals of selective sweeps, we identified a total of seven significant regions that showed signatures of positive selection overlapping or proximal to the highly divergent regions between populations collected from variety A and variety D. These regions are located on chromosomes 1, 2, 6, 7 and 9, spanning from 4.5 to 20 Kb. We further zoomed into these regions to explore candidate genes potentially involved in host variety adaptation. Overall, we identified 26 protein-coding genes (see Supplementary Table S6 online). Among these genes, we identified 24 non-secreted proteins that are predicted to be involved in nutrient transport and metabolism, cell signaling, protein folding and stress response, and nucleotide biosynthesis. In addition, some uncharacterized genes were also identified. These genes may represent potential targets of host-driven selection and may play a role in adaptation of *C. beticola* to variety D. Two genes were also identified that are predicted to encode secreted proteins, which were further predicted to be putative effectors, including one apoplastic effector (EffectorP score: 0.595) and one cytoplasmic effector (EffectorP score: 0.533). To further characterize these two candidate effectors, we performed domain annotation using Pfam HMMER scans and complementary homology searches. Both proteins returned highly significant matches to the cupin superfamily. One effector contained a pirin C-terminal cupin domain, whereas the other matched the pirin domain.

## Discussion

In this study, we analyzed 900 *C. beticola* isolates collected across multiple locations and sugar beet varieties differing in resistance to CLS. To our knowledge, this represents the first large-scale sequencing effort of *C. beticola* in Germany. By combining extensive sampling with location- and variety-specific re-inoculation, we were able to explore patterns of population diversity and test for signals of adaptation associated with both geographic origin and host variety. Such a combination of large-scale sampling, geographic replication, and variety-specific re-inoculation has not previously been applied to *C. beticola*.

By sequencing and analyzing *C. beticola* isolates collected from four locations in Germany in 2022, we established a baseline view of the *C. beticola* population structure in Germany. Overall, populations showed little geographic differentiation, reflecting a largely mixed population. Consistent with previous studies of *C. beticola*^28,43^, the intrinsic genetic diversity of *C*. *beticola* populations is higher compared to other fungal pathogens, such as *Magnaporthe oryzae*^44^ *and Blumeria graminis f. sp. tritici*^45^. We further detected an equal distribution of the two mating types in populations from Einbeck and Oberschneiding that, in conjunction with their high levels of genetic diversity, suggested a potential for sexual reproduction in these two populations. Though no sexual stage has been described for *C. beticola*, this finding aligned with other population studies in *C. beticola* that found equal distribution of the two mating types^3,4,46^. However, in our study, populations collected from Soest and Kösching showed a skewed distribution in the two mating types, potentially supporting a mixed reproductive system with both sexual and clonal reproduction as proposed earlier^47^.

We further performed location-specific re-inoculation across 3 years in three of the four study sites. This resulted in the emergence of diverging lineages in two locations (Soest and Kösching). This finding aligned with the observation of a short-term, within-site differentiation in other *C. beticola* studies across successive years^48^. We assume factors such as local environmental pressures, stochastic processes, or host interactions can foster lineages differentiation. Interestingly, the dispersion was not observed in populations from the Einbeck location, which instead exhibited a genetically stable population structure. We hypothesize that the population might have established Hardy–Weinberg equilibrium prior to the trials. One plausible explanation is that this location serves as a long-term experimental field, which might associate with similar sugar beet varieties and controlled agronomic practices. Such conditions may reduce the impact of genetic drift and selection, which further prevent the formation of location-specific lineages^38^. In addition, although our AMOVA results did not show evidence of local adaptation, the increased variation in the factor “between samples within location” (samples were nested by host variety) over 3 years suggested another factor has driven the dispersion of individuals within locations. In our case, this factor is the host variety.

By performing the variety-specific re-inoculation across 3 years, we imposed high selective pressure especially to populations from the resistant varieties. Variety resistance is known to act as a strong evolutionary force on pathogen populations^11^. Our results showed that host-associated lineages were formed mostly in populations collected from the resistant variety D, suggesting the recurrent selection was specifically acting on these populations. On a resistant variety, fewer successful infections each season followed by re-inoculation from surviving lesions can reduce effective population size and amplify stochastic allele-frequency shifts, even as overall nucleotide diversity declines. These effects are difficult to fully disentangle from selection, but the concurrent rise in pairwise Fst between the populations collected from variety D and other populations, together with signals of selective sweeps in specific genomic regions, supports a contribution of host-driven selection acting on standing genetic variation.

These same sugar beet varieties were also used in our recent ecological study, where resistant variety D consistently delayed disease onset, showed lower disease severity, and produced fewer airborne spores compared to susceptible ones^49^. In contrast, the second resistant variety C only delayed the onset of disease to a limited extent, and this effect was less pronounced when conditions were highly favorable for *C*. *beticola*. We hypothesize that there are differences in the genetic basis of resistance in variety C and D. However, the high level of disease severity observed in this study in the year 2024 demonstrates that artificial inoculation, when combined with favorable infection conditions, can result in poorer differentiation between varieties. Consequently, an increase in background noise could have had a negative impact on the identification of host-driven adaptation in this study. In the antagonistic interactions between hosts and pathogens, hosts play a crucial role in the evolutionary trajectory of pathogens. A recent study in *C. beticola* collected from wild sea beet and cultivated beet elucidated the host specialization in this pathogen^46^. In addition, adaptation driven by varietal resistance was also observed in the oomycete pathogen *Plasmopara viticola*^50^. While our genetic data point to adaptation on variety D, we did not evaluate whether these lineages differ in aggressiveness. Assessing virulence traits such as infection efficiency, lesion development, or sporulation capacity will be crucial in future studies to determine the epidemiological consequences of host-driven divergence. In addition, the rapid emergence of new lineages specific in populations collected from variety D suggests that the adaptation is most likely derived from standing genetic variation that already exist in the populations rather than de novo mutations^51,52^. Preserving such variation to achieve fast adaptation is a common strategy of plant fungal pathogens, enabling rapid shift under selection. For instance, a study on the causal agent of wheat powdery mildew, *Blumeria graminis*, revealed that ancient virulent variants of the AvrPm17 effector were already present in global populations prior to the deployment of the rye-derived Pm17 resistance gene^53^. Moreover, two separate lineages were formed in the population collected from variety D, suggesting the selection pressure may occur on different genetic backgrounds. Future work focused on the genomic comparison between these lineages may identify potential genes that were selected differently.

We further performed a genomic scan in populations collected from variety D at the end of the variety-specific re-inoculation in 2024 to detect possible signatures of selective sweeps. We then associated the results with the highly divergent genomic regions when comparing populations from variety A and D. Genes associated with these regions comprised 26 protein-coding loci, including two putative effectors alongside 24 genes with general physiological functions, such as transporters and metabolic genes, which may be related in enhancing nutrient acquisition under host resistance pressure. Similar findings were addressed in the pathosystem of *Z*. *tritici* and wheat, where genome-wide scans revealed selective sweeps and highly divergent regions enriched for genes involved in host infection, including effector-like functions, secondary metabolite clusters, and transport -related genes, consistent with host-driven selection^54^. In addition, two putative effectors are of particular interest, as effectors are frequently the target of host-imposed selection and play central roles in pathogen adaptation^13^.

Lastly, our population genomic analyses revealed that two candidate effector genes, designated here as CbPir1 and CbPir2, reside within genomic regions showing strong divergence between isolates from resistant and susceptible sugar beet varieties and exhibit evidence of recent selective sweeps. Such signatures suggest that these loci may have been shaped by host-driven selection. Both encode pirin-type domains belonging to the cupin superfamily, whose members share a conserved β-barrel fold with transition metal–binding capacity and redox sensitivity^55,56^. Evidence from fungal systems supports functional roles for pirin homologs in stress biology. For example, the pirin-like protein Prn1 was identified as a key factor in the oxidative stress response in *Candida albicans*^57^. Likewise, pirin-like proteins were shown to contribute to osmotic and oxidative stress tolerance as well as to regulation of patulin biosynthesis in *Penicillium expansum*^58^. Given their predicted localizations, CbPir1 (apoplastic) may act at the host–pathogen interface to influence redox and metal-dependent defenses^59^, while CbPir2 (cytoplasmic) may target intracellular processes linked to redox signaling. Our annotation, combined with the population genetic evidence for host-driven selection, highlighted these two effector candidates for functional validation in understanding the molecular interactions between *C. beticola* and sugar beet varieties.

## Methods

### Experimental field design

Field trials were conducted at four locations in Germany, including Kösching (48°49′00.0"N 11°30′00.0"E), Einbeck (51°49′00.0"N 9°52′00.0"E), Soest (51°34′16.0"N 8°06′33.0"E) and Oberschneiding (48°48′33.5"N 12°38′07.2"E). Four sugar beet varieties bearing different resistant properties provided by project partners that supplied seeds for the trials (A: susceptible; B: susceptible; C: resistant; D: resistant) were cultivated as triplicates in a completely randomized block design. Experimental plots in each trial location were artificially inoculated with CLS-infested leaf materials which were harvested beforehand from the same location in autumn 2021. Each location had their own inoculum bulked as a mixed population. Inoculated plots were separated with isolated plots planted with non-inoculated, resistant varieties to prevent cross-contamination. At the end of the season, leaf materials from each sugar beet variety in each trial location were harvested, dried, and stored separately for the next year’s location- and variety-specific re-inoculation (see Supplementary Fig. S5 online). Plots were inoculated with 4 g/m^2^ of the leaf material blended with semolina in a 1:5 ratio^60^. The same field design and inoculation were repeated in 2023 and 2024 by inoculating the varieties with the location- and variety-specific inoculum collected from the year before. Field management of all trials was conducted according to common agricultural practice without fungicide treatments. Disease severity monitoring of each variety was performed by project partners responsible for each trial location. The results were standardized as KWS rating scale.

### Sample collection and isolation of *C*. *beticola*

In each year, 25 isolates of *C. beticola* were obtained from each variety at each location. This resulted in a population of 100 isolates from each location in each year. Over 3 years, 1200 *C. beticola* isolates were collected from four sugar beet varieties at four different geographical locations in Germany.

To obtain single-conidial isolates, CLS-infected leaf materials were collected at each location by sampling leaves showing 20–30% disease severity with clearly separated lesions. Leaf materials were stored at −20 °C until isolation. To avoid resampling of the same isolate, only one single-conidial isolates was obtained from an individual leaf.

To retrieve single-conidial isolates from the leaf material, lesions were rinsed with 10 µL of water with 2% ampicillin (w/v) by carefully pipetting up and down on the surface of lesions to liberate conidia. The conidia suspension was immediately mixed with 40 µL pre-pipetted water with ampicillin on a water agar plate (1.5% w/v agar in distilled water). This mixture was plated out by a sterile triangular metal rod to equally distribute the suspension. Plates were incubated in the dark at room temperature without sealing for three to 4 days until white, star-shaped colonies were initiated. Plugs containing a single colony were later transferred to Potato Dextrose Agar (PDA) plates with 2% (w/v) streptomycin for subsequent cultivation. The sub-cultivation process was repeated until no contamination occurred. Mycelium plugs with *C. beticola* were transferred to PDA plates for stationary growth. After approximately 2 weeks, overgrown mycelium plugs from each isolate were transferred to potato dextrose broth (PDB) medium. Fungal materials were harvested from PDB material seven to 10 days after inoculation for DNA extraction. Harvested fungal materials were freeze-dried (Alpha 1–4 LSCbasic, Martin Christ Gefriertrocknungsanlagen GmbH, Osterode am Harz, Germany) overnight and stored at −20 °C until DNA extraction.

### DNA extraction and species confirmation

DNA extraction was conducted with the DNeasy Plant Mini Kit (Qiagen, Hilden, Germany) following the manufacturer’s instructions with a final elution volume of 60 µL. Approximately 25 mg of starting material was used for fungal DNA extraction for an optimal quality and quantity. DNA samples were verified through a spectrophotometer and gel electrophoresis to ensure DNA integrity.

Polymerase chain reaction (PCR) assays were performed after DNA extraction to ensure all the prepared isolates are derived from *C. beticola*. For this, a *C.* *beticola*-specific primer pair was used from the study of Knight and Pethybridge^61^, targeting the calmodulin gene in *C. beticola* with a fragment size of 199 bp. Each 20 µL-reaction contains ten µL of DreamTag PCR Master Mix (Thermo Fisher Scientific Inc.), one µM of each primer (forward: 5’ GGCAGAGCTAACGACAGCAA 3’; reverse: 5’ TTGTTGTCGGCGTCGACT 3’), one µL of the template DNA and seven µL DEPC-treated water. The programme was as follows: initial denaturation at 95 °C for 3 min; 35 cycles of denaturation at 95 °C for 30 s, annealing at 64 °C for 30 s, and extension at 72 °C for 15 s; and a final extension at 72 °C for 5 min.

### Library preparation and sequencing

Library preparation was performed using a MagicPrep™ (Tecan Trading AG, Switzerland) with the Revelo DNA-Seq Enz for MagicPrep NGS, kit A (Tecan Trading AG, Switzerland) upon manufacturer’s protocol. Quantity and quality control of libraries were performed via Qubit 4 fluorometer (Thermal Fisher Scientific Inc, USA) and TapeStation Systems (Agilent Technologies, Inc, CA, USA). Whole genome sequencing was conducted by Novogene Co, Inc (CA, USA) on platform NovaSeq™6000 or HiSeq™2500 (Illumina, Inc.) depending on the availability, using paired end 150 bp reads.

### Mapping and variant calling

Sequencing data was downloaded from Novogene to the United States Department of Agriculture high performance computing cluster SciNet. Sequencing read quality was assessed using FastQC (Andrews^62^) and trimmed using Trimmomatic v0.36 to remove adapter and low-quality sequences (Bolger et al.^63^). Trimmed reads were aligned to the *C. beticola* isolate Cb09-40 reference^64^ using the program BWA-MEM^65^. Variants were identified using bcftools “mpileup” and “call” with ploidy set to one for haploid calls^66^. Variants were subsequently filtered to retain high quality variants by removing variants with less than 3 supporting reads and a minimum quality score less than 20. Two variant files were produced, one having all sites present, referring as an “all-site dataset”, regardless of a variant being present and a variant file with only variants represented, referring as a “full variant dataset”.

### Quality control and filtering of the VCF file

R package *vcfR* (version 1.15.0)^67^ was used to read VCF files and visualize the Phred-scaled quality of the variants. The flag *ploidy* was set to “1”. Additional filtering steps were applied for various analysis to satiate the analysis assumptions. For analysis requiring unlinked loci, variant files were filtered to remove any variants within 2 Kb of one another, referring as the “independent SNP dataset”, as previously performed for *C. beticola* in Taliadoros et al.^46^ using the VCFtools software package (version 0.1.17, with additional “haploid” fork)^68^ to acquire unlinked SNP data for further clustering analysis.

### Genetic diversity, population divergence and neutrality

Genome-wide nucleotide diversity (π), absolute nucleotide divergence (Dxy) and Weir and Cockerham’s Fst^69^ were calculated with the software pixy^70^. The window size was set to be 10 Kb for the sliding window calculation with default settings. To assess the robustness of the mean pairwise Fst estimates, 95% confidence intervals were calculated by bootstrap resampling of genomic windows (1000 replicates with replacement) within each population pair and year. Genome-wide Tajima’s D^17^ was calculated by VCFtools (version 0.1.17, with additional “haploid” fork)^68^ with a window size of 10 Kb. Global π and Tajima’s D of each population were further calculated by taking the mean values of the genomic scan and the significance of the differences (*p* < 0.05) was examined by the Kruskal–Wallis test followed by a post-hoc pairwise Wilcoxon test. Visualization was achieved using the R package *ggplot2* (version 3.5.0)^71^ and ggpubr (version 0.6.0)^72^. The software BEDTools (version 2.18)^73^ was used to identify overlapped regions from various statistics. More specifically, the regions that exhibited the most significant divergence between populations A and D were identified by overlapping regions that had the top 5% highest Dxy and Fst values. These regions were further referred to as “the highly divergent regions”.

### Population structure and distance tree

To perform principal component analysis (PCA), the “independent SNP dataset” was initially converted to a *genlight* object with the ploidy flag set to be “1”. PCA was performed using the function *glPCA()* in package *adegenet* (version 2.1.10)^74^. R package *ggplot2* (version 3.5.0)^71^ was used for PCA visualisation. In addition, bitwise genetic distances were calculated using *bitwise.dist* function in the poppr R package^75^. An unweighted pair group method with arithmetic mean (UPGMA) tree was generated using the aboot() function, with 100 bootstrap replicates and a support cutoff of 50% to assess branch stability. The visualization of the tree was achieved with ggtree package (version 3.4.2)^76^.

### Mating types

The distribution of the *MAT1-1-1* gene (GenBank accession number: DQ264736) of each population was studied in CLC Genomics Workbench (version 23.0.4, Qiagen, Aarhus A/S, Denmark). More specifically, the genomic coordinates of *MAT1-1-1* in the reference genome were first identified, and the BAM file of each isolate was then imported and mapped with default settings to the reference genome as indicated above. Isolates showing mapped reads spanning the *MAT1-1-1* locus were classified as MAT1-1-1, whereas isolates lacking mapped reads across this locus were classified as MAT1-2-1. A Chi-square goodness-of-fit test was used to evaluate whether the observed frequencies of the two mating types deviated significantly from an expected 1:1 ratio within each population. R package *ggplot2* (version 3.5.0)^71^ was used to visualise the results.

### Genomic scans to detect selective sweeps

To further identify potential signatures of positive selection, we performed a genomic scan by software RAiSD with default window size^77^ on population collected from resistant variety D. To account for the effect of demographic history on sweep detection, we used the population from the susceptible variety A as a neutral reference. Chromosome-specific thresholds were defined as the 99.5th percentile of the μ statistics from population from variety A, under the assumption that selection pressure in this population is minimal due to host susceptibility. These thresholds were applied to population from variety D, and only windows with μ statistics exceeding the chromosome-specific cutoff were retained as candidate sweep regions. These regions were further overlapped with the highly divergent regions between the two populations. Regions flanking 50 Kb of the highly divergent regions that had selective sweeps detected were kept as the final significant regions to screen the key genes that were under selection. Subsequently, candidate genes within the regions were further extracted from the gene annotation list. Among them, putative secreted proteins were identified based on a signal-peptide using SignalP^78^. Furthermore, the secreted proteins were tested in EffectorP v.3.0^79^ to predict putative effectors. Predicted protein sequences of candidate effectors were scanned for conserved domains using the Pfam database^80^ with the HMMER web server and default parameters.

## Supplementary Information

Supplementary Information 1.

Supplementary Information 2.

## Data Availability

Sequencing reads for all isolates collected have been deposited at NCBI-SRA under bioproject PRJNA 1,311,077. Supplementary file 1 contains isolates specific coverage data with accession and biosample identifiers included.
