# Supplementary material for: Experimental evolution reveals genomic signatures of variety-specific selection of Cercospora beticola in Germany
Source: Sci Rep. 2026 May 21;16:15881. doi: 10.1038/s41598-026-52994-7 (PMC13195098; doi:10.1038/s41598-026-52994-7)
Supplement: Supplementary file 1 — Supplementary Information 1. [file 41598_2026_52994_MOESM1_ESM.pdf]

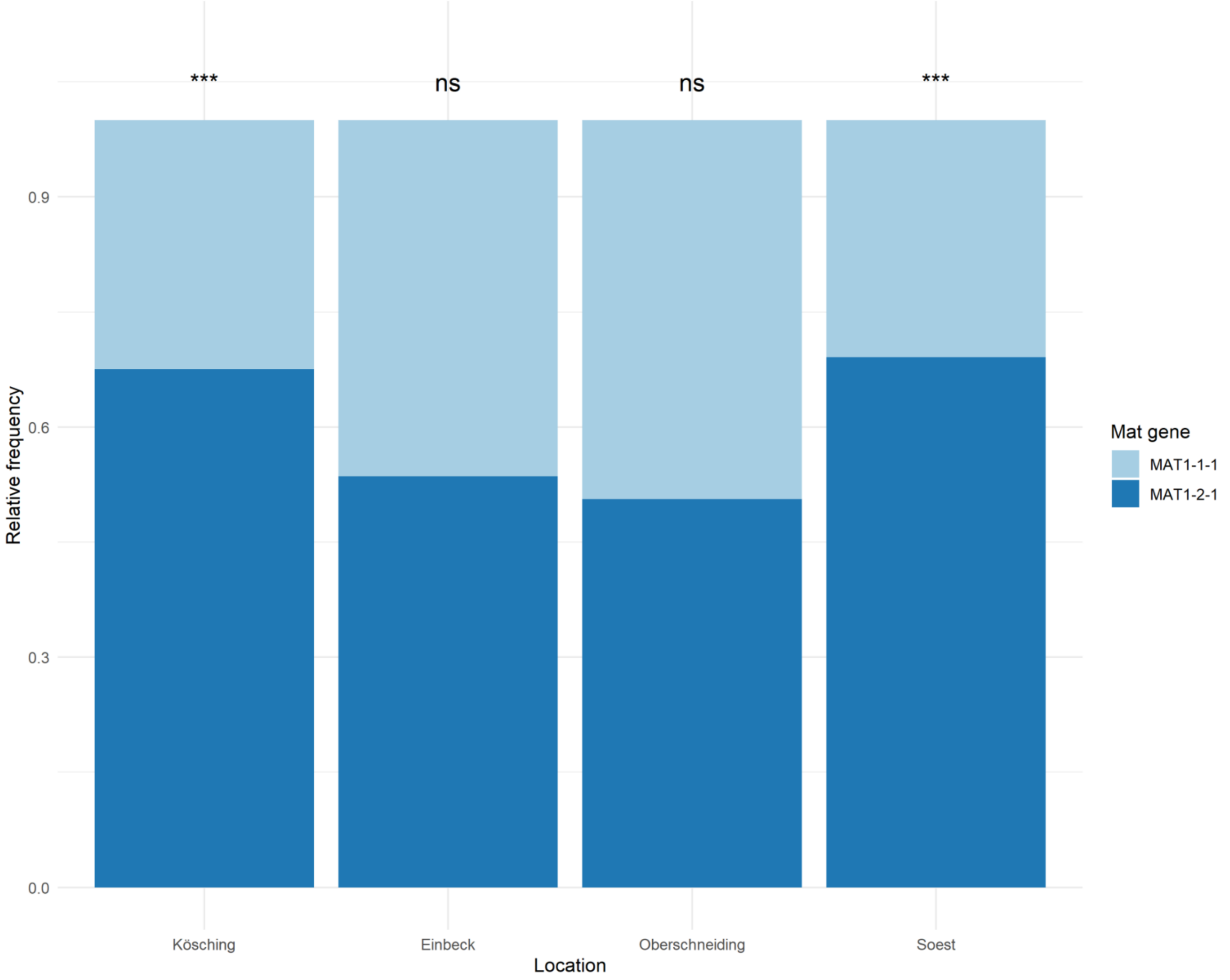

Supplementary Figure S1. Relative frequencies of the two mating types in each *C. beticola* population collected from four different trial locations in 2022. The deviation of a 1:1 distribution was tested in each population using a chi-square test. Significant deviations from the expected ratio are indicated by asterisks (p < 0.05).

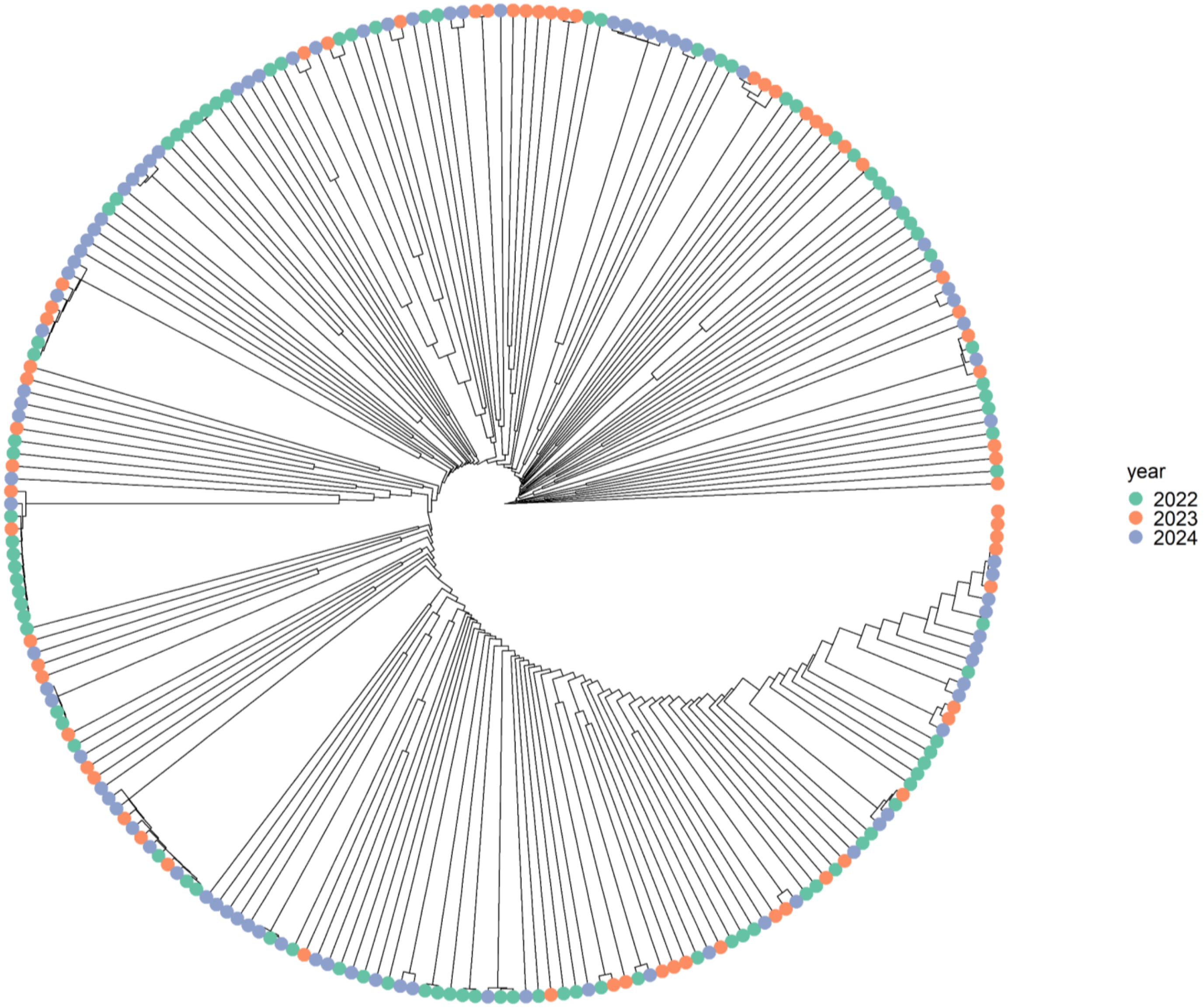

Supplementary Figure S2. Circular UPGMA distance tree based on pairwise bitwise genetic distances among all isolates from location Einbeck. Tip colors indicate the year of isolation (2022–2024).

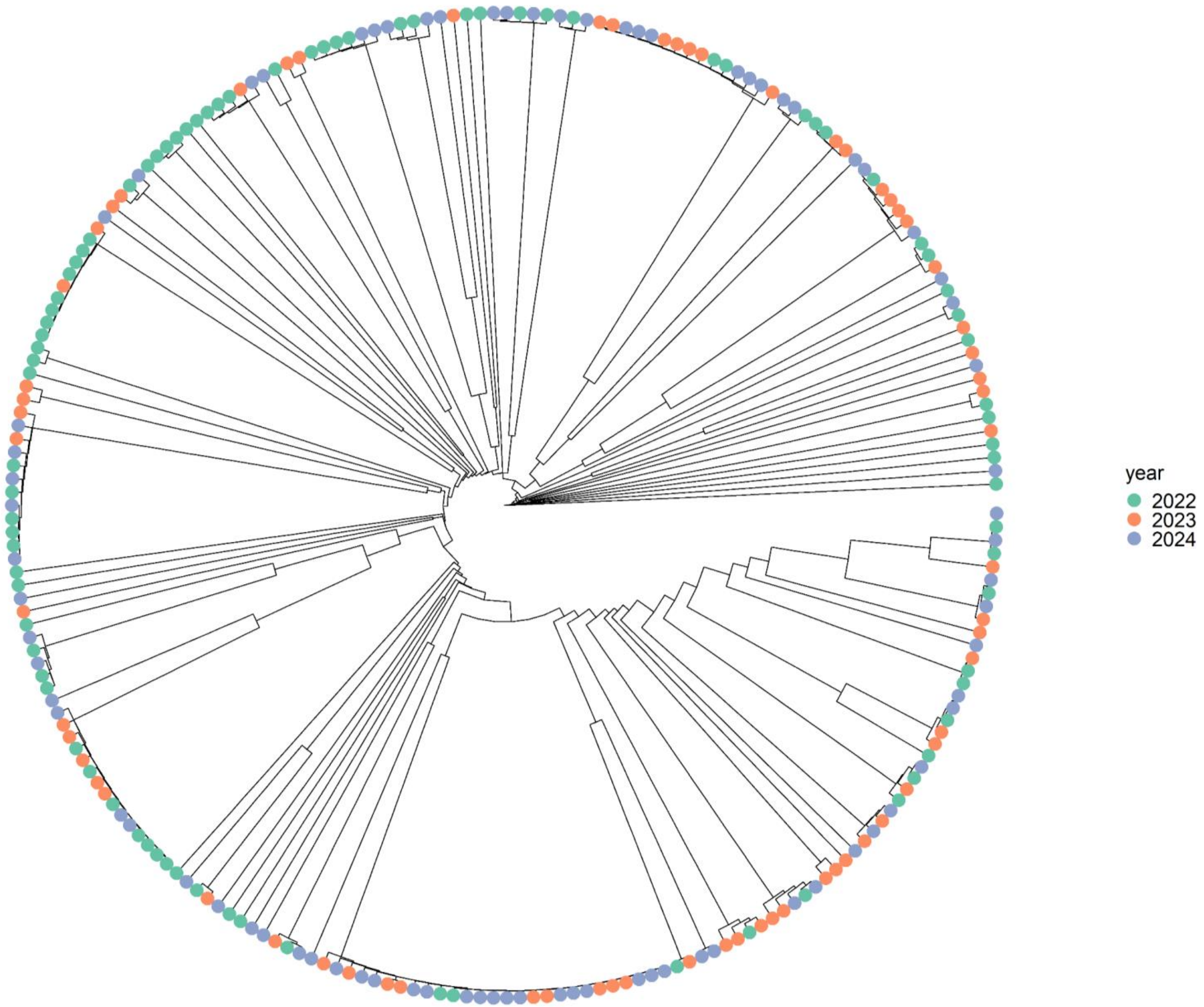

Supplementary Figure S3. Circular UPGMA distance tree based on pairwise bitwise genetic distances among all isolates from location Soest . Tip colors indicate the year of isolation (2022–2024).

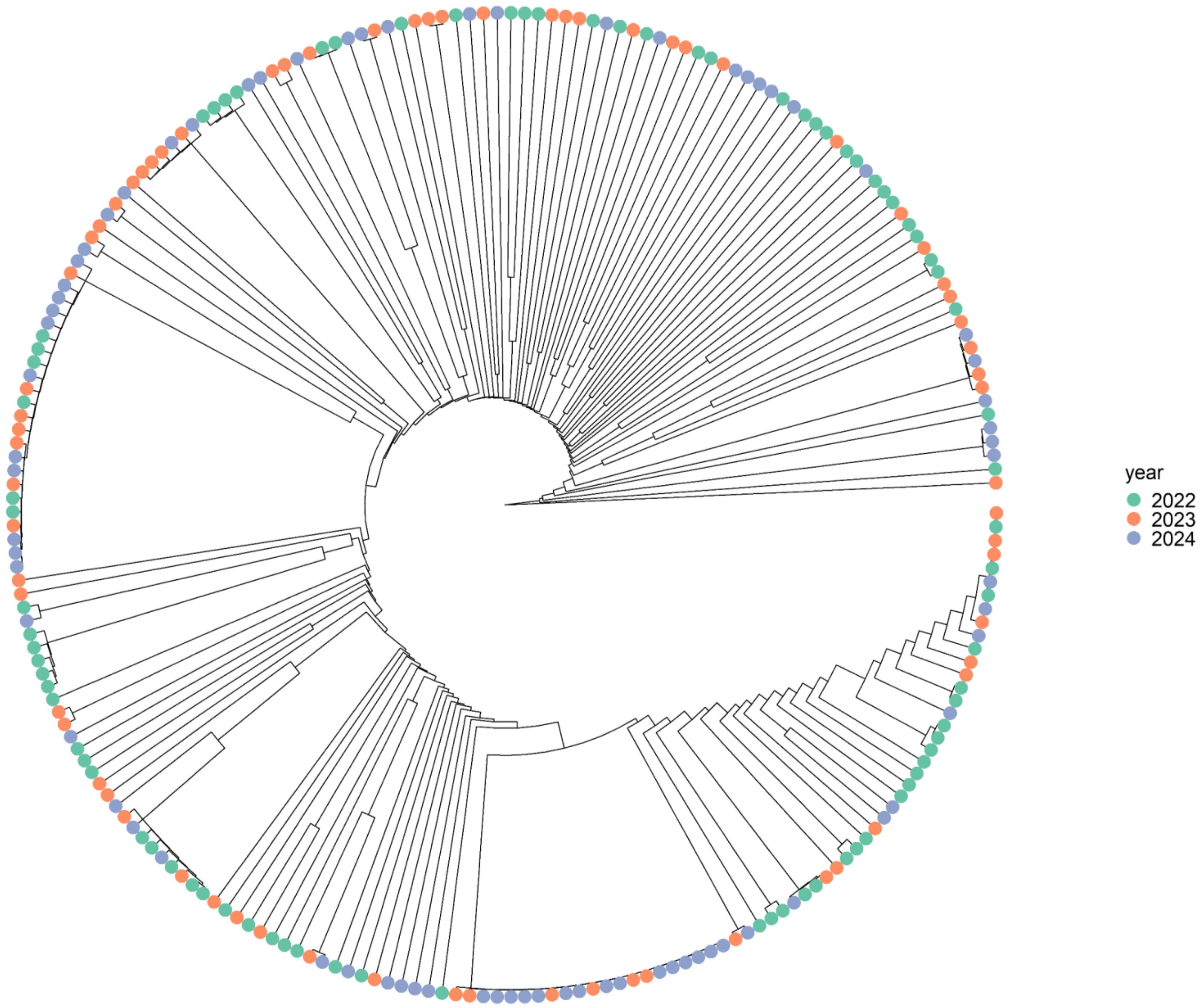

Supplementary Figure S4. Circular UPGMA distance tree based on pairwise bitwise genetic distances among all isolates from location Kösching. Tip colors indicate the year of isolation (2022–2024).

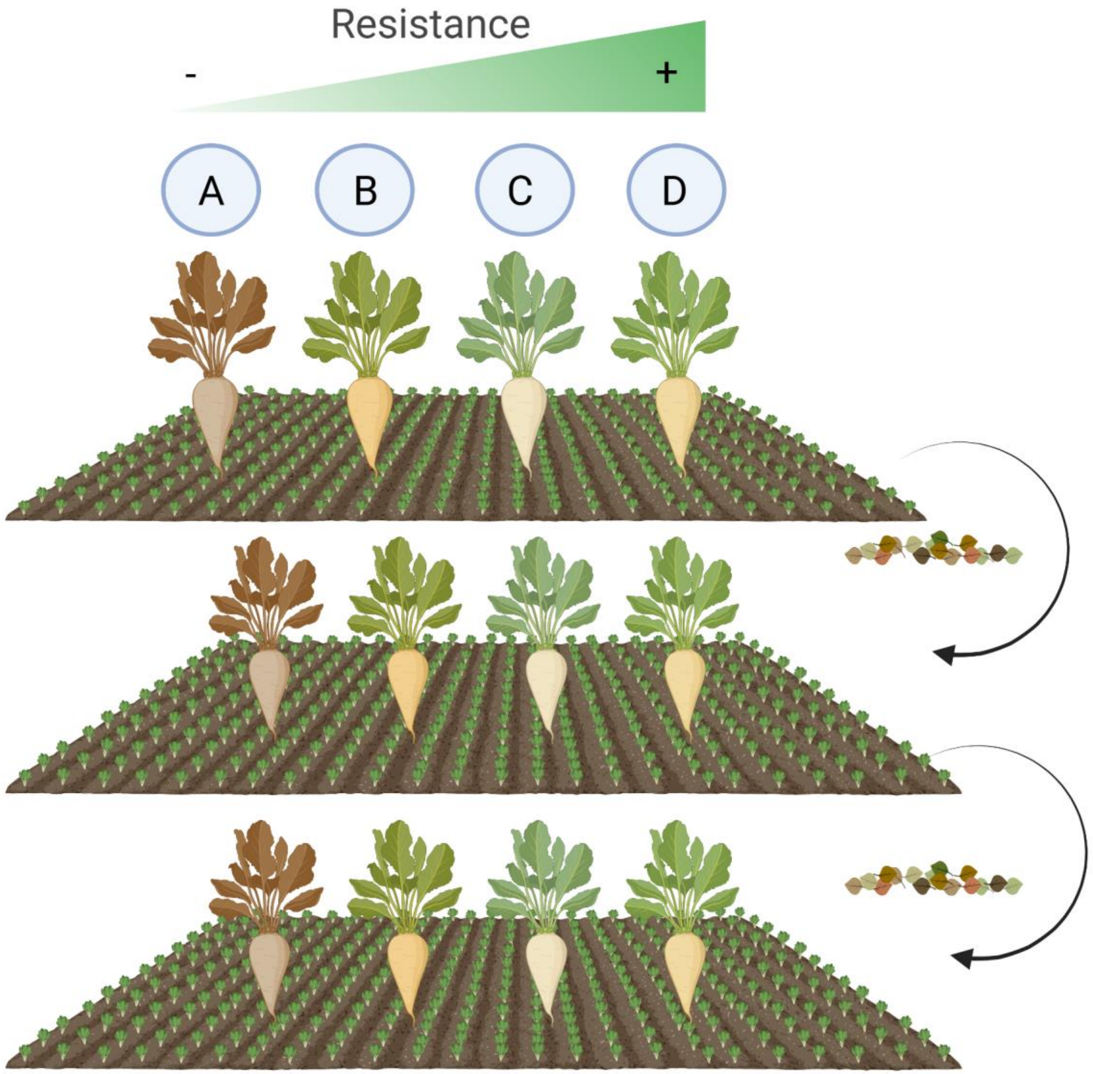

Supplementary Figure S5. Schematic illustration of the field experimental setup. Four sugar beet varieties with different resistance properties were inoculated with *Cercospora beticola*. At the end of each season, infected leaf material was collected separately from each variety and used as inoculum for the subsequent season. Leaf material collected from a given variety was used exclusively to inoculate the same variety in the following season, a so-called variety-specific reinoculation.

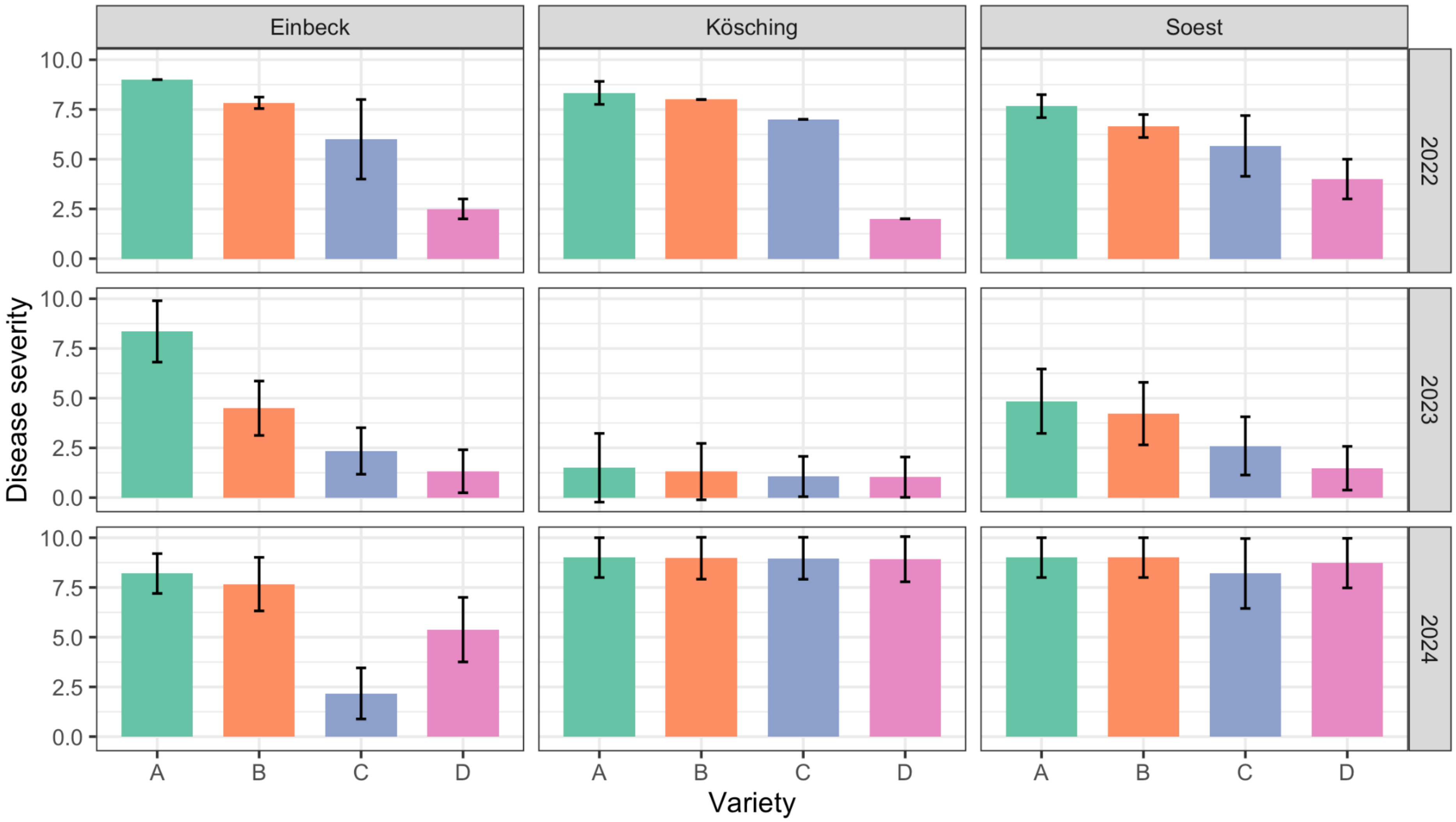

Supplementary Figure S6. Disease severity of CLS on each variety in three reinoculated locations across three years. Bars represent mean disease severity with standard deviation.
